# Supplementary material for: Modeling glioblastoma heterogeneity as a dynamic network of cell states
Source: Mol Syst Biol. 2021 Sep 16;17(9):e10105. doi: 10.15252/msb.202010105 (PMC8444284; doi:10.15252/msb.202010105)
Supplement: Supplementary file 6 — Source Data for Figure 5 [file MSB-17-e10105-s004.zip › Figure5A_sourcedata/GSEA_3017/nosigdb_stateC.GseaPreranked.1622795002785/SEURAT_CELLCYCLE.html]

Details for gene set SEURAT\_CELLCYCLE[GSEA]

|  || Dataset | state13017 |
| Phenotype | NoPhenotypeAvailable |
| Upregulated in class | na\_pos |
| GeneSet | SEURAT\_CELLCYCLE |
| Enrichment Score (ES) | 0.7351658 |
| Normalized Enrichment Score (NES) | 4.068672 |
| Nominal p-value | 0.0 |
| FDR q-value | 0.0 |
| FWER p-Value | 0.0 |
Table: GSEA Results Summary

  

Fig 1: Enrichment plot: SEURAT\_CELLCYCLE      
 Profile of the Running ES Score & Positions of GeneSet Members on the Rank Ordered List

  

| PROBE | GENE SYMBOL | GENE\_TITLE | RANK IN GENE LIST | RANK METRIC SCORE | RUNNING ES | CORE ENRICHMENT || 1 | ATAD2 |  |  | 7 | 0.649 | 0.0455 | Yes |
| 2 | CCNE2 |  |  | 15 | 0.570 | 0.0839 | Yes |
| 3 | CLSPN |  |  | 17 | 0.561 | 0.1322 | Yes |
| 4 | RRM2 |  |  | 19 | 0.542 | 0.1787 | Yes |
| 5 | HMGB2 |  |  | 20 | 0.534 | 0.2263 | Yes |
| 6 | GMNN |  |  | 38 | 0.453 | 0.2367 | Yes |
| 7 | UHRF1 |  |  | 45 | 0.436 | 0.2650 | Yes |
| 8 | RAD51AP1 |  |  | 46 | 0.435 | 0.3038 | Yes |
| 9 | PCNA |  |  | 48 | 0.435 | 0.3407 | Yes |
| 10 | TMPO |  |  | 49 | 0.432 | 0.3793 | Yes |
| 11 | DTL |  |  | 50 | 0.430 | 0.4176 | Yes |
| 12 | TOP2A |  |  | 52 | 0.426 | 0.4538 | Yes |
| 13 | WDR76 |  |  | 56 | 0.403 | 0.4844 | Yes |
| 14 | TYMS |  |  | 60 | 0.390 | 0.5139 | Yes |
| 15 | RRM1 |  |  | 62 | 0.386 | 0.5465 | Yes |
| 16 | NASP |  |  | 75 | 0.371 | 0.5585 | Yes |
| 17 | POLD3 |  |  | 76 | 0.367 | 0.5912 | Yes |
| 18 | SMC4 |  |  | 78 | 0.365 | 0.6220 | Yes |
| 19 | GINS2 |  |  | 83 | 0.360 | 0.6470 | Yes |
| 20 | USP1 |  |  | 92 | 0.352 | 0.6643 | Yes |
| 21 | FEN1 |  |  | 98 | 0.342 | 0.6860 | Yes |
| 22 | NUSAP1 |  |  | 100 | 0.340 | 0.7145 | Yes |
| 23 | SLBP |  |  | 106 | 0.331 | 0.7352 | Yes |
| 24 | HELLS |  |  | 171 | 0.271 | 0.6468 | No |
| 25 | POLA1 |  |  | 179 | 0.266 | 0.6582 | No |
| 26 | MSH2 |  |  | 180 | 0.265 | 0.6818 | No |
| 27 | MCM4 |  |  | 189 | 0.259 | 0.6908 | No |
| 28 | CDK1 |  |  | 191 | 0.255 | 0.7118 | No |
Table: GSEA details [plain text format]

  

Fig 2: SEURAT\_CELLCYCLE: Random ES distribution      
 Gene set null distribution of ES for **SEURAT\_CELLCYCLE**
